# Monitoring and Evaluating Progress towards Universal Health Coverage in Tunisia

**DOI:** 10.1371/journal.pmed.1001729

**Published:** 2014-09-22

**Authors:** Mohamed Kouni Chahed, Chokri Arfa

**Affiliations:** 1 Department of Epidemiology and Public Health. Faculty of Medicine of Tunis, Tunis, Tunisia; 2 National Institute of Labor and Social Studies, University of Carthage, Tunis, Tunisia

## Abstract

This paper is a country case study for the Universal Health Coverage Collection, organized by WHO. Mohamed Kouni Chahed and colleagues illustrate progress towards UHC and its monitoring and evaluation in Tunisia.

*Please see later in the article for the Editors' Summary*

This paper is part of the PLOS Universal Health Coverage Collection. This is the summary of the Tunisia country case study. The full paper is available as Supporting Information file Text S1.

## Background

The World Health Organization defines universal health coverage (UHC) as a situation in which all people who need health services receive them, without incurring financial hardship. UHC is currently perceived as a crucial component of sustainable development and listed as one of the possible goals of the post-2015 development agenda.

Very soon after its independence in 1956, Tunisia made health care free for all through a completely government-funded system. In 1982, the country decided to implement a large network of primary health care centers. Given these steps, Tunisia made substantial progress in improving access to health care. Currently, health care is delivered both by an extensive public health care facilities network and a growing private sector.

## Universal Health Coverage: The Policy Context

Tunisia had built a national health system and, over the past 30 years, made particular efforts in developing the health workforce and rehabilitating facilities. However, the remaining gap of access to health services between poor populations and areas and those with better living conditions contributed to the emergence of 2011 revolution. Since 2011, population and civil society have been demanding new health policy and approaches to track remaining gaps and to ensure equity.

In terms of financial risk protection, the government of Tunisia has been implementing a two-tiered social protection system with health insurance and subsidized or free care. Based on the principles of assistance and insurance, it is funded through employee contributions and government-subsidized coverage for the poorest sectors of the population.

## Monitoring and Evaluation for UHC

The main source of data for the health sector is routine administrative health facilities data, which are used to produce some health indicators in the form of service utilization or coverage rates by human resources of health and facilities, by district and region. Periodic population-based surveys, (Demographic and Health Survey) and the Multiple-Indictor Cluster Survey (the last was conducted in 2011), are used to evaluate health interventions coverage and maternal and child mortality rates. The National Institute of Statistics uses periodic household surveys to assess the living standards of households and provides disaggregated health expenditures data by wealth quintiles.

Although a large amount of data and sources of data are available and may be used to assess and evaluate progress towards UHC in Tunisia, there is no comprehensive means within the health information system for its monitoring since no UHC-related core indicators have been defined.

## Progress towards UHC in Tunisia

Trends of many health indicators during the past three decades show that Tunisia has made substantial progress in coverage indicators. Coverage rates for antenatal and postnatal care, attended deliveries, and immunizations have improved nationwide following implementation of maternal and child health interventions within the basic health care network [1],[2].

**Figure 1:**
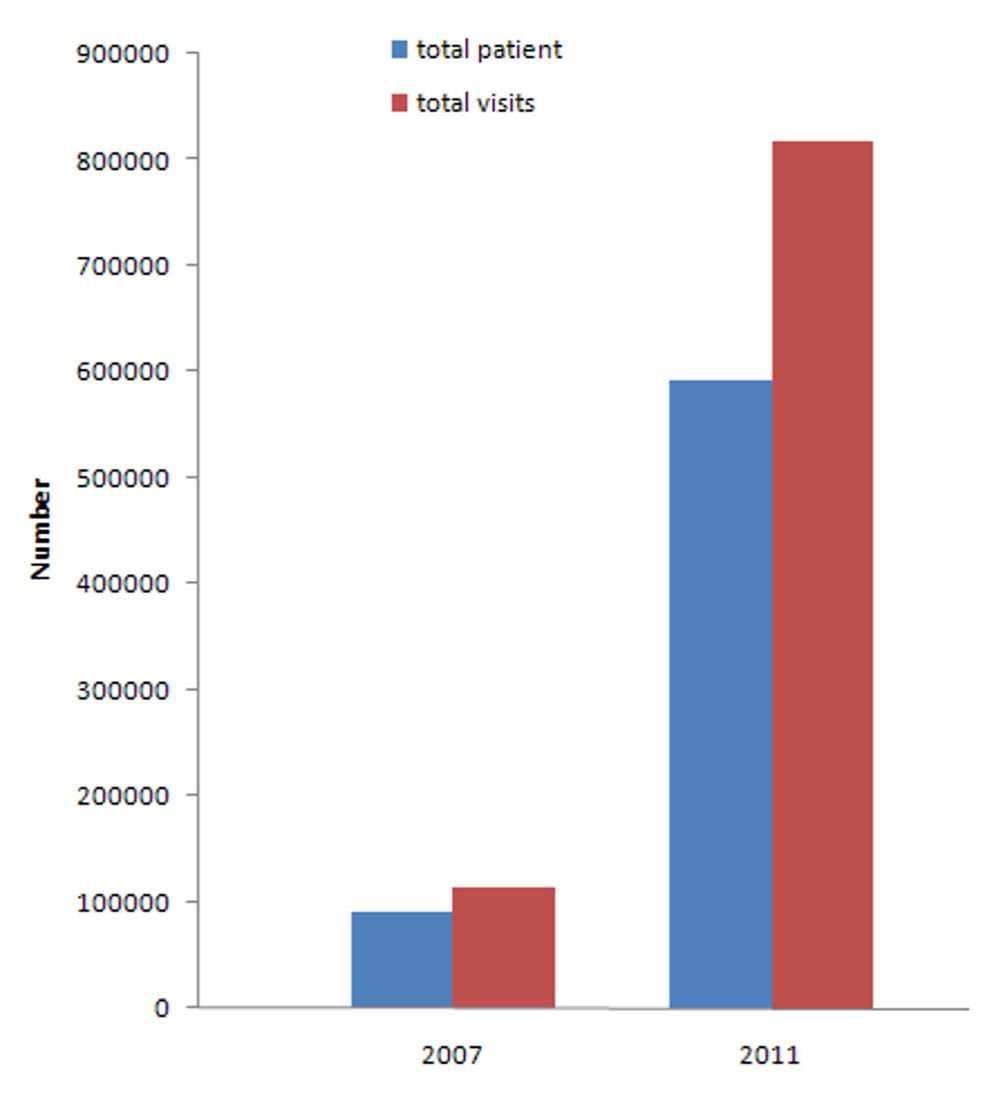
Chronic disease (hypertension and diabetes) trends [6].

Tunisia seems to be on schedule to meet Millennium Development Goals (MDGs) targets without further policy efforts, with the exception of maternal mortality. Until 2008, progress in reaching the target of reducing maternal mortality by two-thirds was insufficient and it remains one of Tunisia's largest MDG challenges. On the other hand, Tunisia has an ageing population and the global burden of disease has shifted from infectious diseases and a focus on maternal and child health to chronic conditions and injuries, therefore new demands will arise that will need to be addressed (Figure 1) [3],[4].

Health equity remains an important issue in Tunisia. Ample evidence reveals the persistent gaps between the coastal areas and the western part of the country, where the number of specialists and doctors, equipment quality, and service coverage are all much lower. In terms of drug availability, while the health system tries to maintain adequate stocks of medicine, the likelihood of shortages is common in all health facilities.

Despite the existence of two insurance schemes, insurance coverage in Tunisia remains incomplete— 8% to 10% (nearly one million people) are not covered. In addition, there is consensus that the public medical aid schemes for the poorest sectors of the population are inefficient, and place a large strain on public hospitals' budgets. The Tunisian population also has a high average household spending on health care; out-of-pocket expenditure was as high as 45% of total health expenditure in 2010. Tunisian citizens in the low income bracket are not, therefore, guaranteed access to the health care they need despite existing efforts [5].

## Conclusions and Recommendations

Tunisia has made substantial progress toward achieving UHC. However, its health system currently faces some obstacles: the remaining gap in access to health services between poor populations and those with better living conditions; the emergence of chronic and non-communicable diseases that require growing resources to make needed treatments available; the unbalanced development of the health system with a growing private sector contrasting with a less efficient institutional public health sector; and the remaining high level of out-of-pocket expenses.

Finally, Tunisia needs to implement specific UHC in-country monitoring mechanisms including relevant tools to measure progress in equity and financial risk protection among the different wealth quintiles and geographical areas.

## Supporting Information

Text S1 **The full country case study for Tunisia.**
(DOCX)

## Abbreviations

UHC: universal health coverage
