# Supplementary material for: Monitoring and Evaluating Progress towards Universal Health Coverage in Tunisia
Source: PLoS Med. 2014 Sep 22;11(9):e1001729. doi: 10.1371/journal.pmed.1001729 (PMC4170955; doi:10.1371/journal.pmed.1001729)
Supplement: Text S1 — The full country case study for Tunisia. (DOCX) [file pmed.1001729.s001.docx]

**Full Case Study: Monitoring and evaluating progress towards Universal Health Coverage in Tunisia**

Mohamed Kouni Chahed^1^ and Chokri Arfa^2^

^1^ Department of epidemiology and public health. Faculty of Medicine of Tunis. Tunis, Tunisia

^2^ National Institute of Labor and Social Studies. University of Carthage. Tunisia

*Corresponding author: Mohamed Chahed

Email: mchahe@yahoo.fr

**This paper is the full country case study to accompany the summary paper “Monitoring and evaluating progress towards Universal Health Coverage in Tunisia” that is part of the Universal Health Coverage Collection. Not commissioned; externally reviewed.**

**Abstract:** Very soon after its independence, in 1956, Tunisia made health care free for all through a completely government-funded system. In 1982, the country decided to implement a large network of primary health care centers. Currently, the Health care delivery is assumed both by an extensive public health care facilities network and a growing private sector.

Regarding Health risk protection, The Tunisia’s government has been implementing a social protection system with two-tiered components of health insurance and subsidized or free care. It’s funded through employee contributions and government-subsidized coverage for those who are poor and pro-poor population.

Tunisia would seem on track to timely meeting the MDGs targets without further policy efforts, only for maternal mortality. Until 2008, progress in achieving the target of reducing by two thirds of maternal mortality was being insufficient and remains one of the biggest MDG challenges. In the other side, Tunisia is currently going to aging and there’s a shift in the global burden of disease from infectious diseases and maternal and child health conditions to chronic conditions and injuries and new demands will be made and need to be addressed.

Given this, Tunisia made a significant progress to achieve UHC. However, the Health system faces currently some obstacles: The remaining gap of access to health services between poor populations and those with better living conditions; the emergence of chronic and non-communicable diseases which require lot resources to make available needed treatments; the unbalanced development of the health system with a growing private sector contrasting with an institutional health sector less efficient; and the remaining high level of Out-of-pocket expenses.

Although Tunisia has to implement specific UHC in-country monitoring mechanisms including relevant tools to measure progress in equity and financial risk protection among the different wealth quintiles and geographical areas.

**Summary Points:**

1. Over the past 50 years, Tunisia had built a national health system and made continued efforts in developing the health workforce and rehabilitating facilities in order to improve access to health.

2. Since 1960, Tunisia had also implemented a social protection system with two-tiered components of health insurance and subsidized or free care. Based on the principles of assistance and insurance, it’s funded through employee contributions and government-subsidized coverage for those who are poor and pro-poor population.

3. Trends of many health indicators during the last three decades show that Tunisia made a big progress to achieve UHC and would seem on track to timely meeting the MDGs targets without further policy efforts, only for maternal mortality.

4. However, health inequity remains as an important challenge as many evidences point out the persisting gaps between the coastal areas and those of western part of the country, where the number of specialists and doctors, the quality of equipment and the coverage of services are all much lower.

5. There’s also an unbalanced development of the health system with a growing private sector contrasting with a less efficient public health sector, incomplete health insurance coverage ( about 10% are not covered) and remaining high level of Out-of-pocket expenses.

6. Tunisia has to implement specific UHC in-country monitoring mechanisms including relevant tools to measure progress in equity and financial risk protection among the different wealth quintiles and geographical areas.

**1. Background**

The World Health Organization defines Universal Health Coverage (UHC) as a situation in which all people who need health services receive them, without incurring financial hardship [1]. UHC is currently perceived as a crucial component of sustainable development and listed as one of the possible goals of the post-2015 development agenda.

Very soon after independence, in 1956, Tunisia made health care free for all through a completely government-funded system. In 1960, Tunisia had also implemented a social protection system with two-tiered components of health insurance and subsidized or free care. Based on the principles of assistance and insurance, it’s funded through employee contributions and government-subsidized coverage for those who are poor and pro-poor population. In 1982, the country decided to implement a large network of primary health care centers. Given this, Tunisia made a big progress in order to improve access to health care. Currently, the Health care delivery is assumed both by an extensive public health care facilities network and a growing private sector.

Tunisia, as many countries through the world, has declared its commitment to achieve universal health coverage (UHC) since 2010. However, a revolution had happened in 2011 [2] and nothing was done to introduce policies and approaches aiming to achieve the goal to track gaps of the Tunisia’s healthcare system which is leaving many inadequately covered. Through a very broad overview of the health situation in Tunisia including coverage for preventive interventions, as well as institutional deliveries, health impact indicators and financial risk protection analysis, the paper aims to bring out these issues clearly and discuss how UHC could be monitored in Tunisia.

**2. Universal health coverage: the policy context**

Tunisia is a North African country, situated between Algeria at west and Libya at east and with a long Mediterranean coastline in the north and the east. During the last three decades, Tunisia has undergone a steady development in the context of a market oriented economy. With a gross domestic product by capita of 4.351$ US (2012), it is ranked 94th on the Human Development Index scale in 2013 (0.712). But this upper middle level of human development is unevenly distributed, higher in the main cities and in the eastern coastal regions.

The 2011 revolution has served to expose the structural weaknesses of the Tunisian economy. Unemployment rose from 13% in 2010 to above 18% as of early 2012 [3]. The national average unemployment level for women reached 27.4% and the national level for those with tertiary qualifications reached 29.2%. Disaggregated by region, the highest unemployment rates were recorded in the south area, i.e., 29.5% in the south-west and 27.2% in the south-east. The lowest rates were recorded in the north-east (14.5%) and the mid-east (11.9%). Tunisia has long exhibited a significant divide between rural and urban areas, and especially between the developed coast and the poorer interior. At least half of the population lives in Tunis and coastal towns, and there is population drift toward these areas.

Over the past 50 years, Tunisia had built a national health system and made lots efforts in developing the health workforce and rehabilitating facilities. Currently, Tunisia has an extensive public health care facilities network with 2091 primary health care centers (1 center per 5105 inhabitant) [4], 118 district hospitals and rural maternities, 33 regional hospitals providing secondary care and 24 academic hospitals (specialized care and teaching hospitals) which means 1.8 hospital bed per 1000. The private sector is the second provider of Health care in Tunisia. It runs among the whole country near 3000 general physician’s cabinets, more than the number of GP working in the institutional health centers, about 3300 specialized physician’s cabinets and 80 inpatient clinics. The private sector is more developed in the coast part than the western part of the country.

The total health expenditures were estimated at about 2.7 million Dollars in 2010, indicating a per capita health expenditure of 350 USD (Table S2). The health financing was largely supported, till the end of the 1980s, by the governmental budget and the Social Security Funds (SSF) with about 65% of total health expenditures (THE) being funded from both fiscal and social contributions. Since the 1990s, total public contribution has decreased to 52% following the financial crisis during the second half of the 1980s and the subsequent adjustment programs that impacted government budgets. Such reduction induced a significant rise of household direct expenditures. In 2010, total health expenditures are respectively financed through general government revenues (25.9%); National Fund of Health Insurance (NFHI) contributions (28.2%); private insurance premiums (4.5%), and out-of-pocket payments (40.3% of which 81% was paid for private providers). External sources of financing were insignificant representing less than 0.1% of total health expenditures (Table S3).

| **Ratio** | **1995** | **2000** | **2005** | **2010** |
| --- | --- | --- | --- | --- |
| **Total Health Expenditures (THE) in Million of Tunisian Dinars (TD)*** | 1 048.32 | 1581.2 | 2247.3 | 4019.3 |
| **Share THE/ Gross Domestic Product (%)** | 5.5 | 5.5 | 5.9 | 6.3 |
| **General government expenditure on health (GGHE) as % of THE** | 51.7 | 52.7 | 50.6 | 54.9 |
| **GGHE as % of General government expenditure** | 8.2 | 8.1 | 9.8 | 10.7 |
| **Social security funds as % of GGHE** | 24.9 | 32.2 | 45.7 | 47.7 |
| **Out of pocket expenditure (OOP) as % of PvtHE** | 78.4 | 84.2 | 86.8 | 89.8 |
| **Share OOP/ THE (%)** | 37.9 | 44.4 | 42.9 | 40.5 |
| **THE per capita (Tunisian Dinars)** | 130.0 | 164.1 | 214.4 | 382.8 |
| **External resources on health as % of THE** | 0.9 | 1.1 | 0.5 | 0.1 |

*1 USD = 1.55 TD

**Table S2:** Selected indicators for expenditures on health (1995-2010)

Data Sources: NHA 2002, 2005, NIS 2013, WHO 2010 and author estimation

| **Source of Health Financing** | **% of Total** | |
| --- | --- | --- |
| Government Spending (Budget) |  | **25,9%** |
| **NFHI** |  | **28,2%** |
| Share of private sector employees | **15,7%** |  |
| Share of public sector employees | **12,5%** |  |
| **Households** |  | **45,8%** |
| Share of OOP | **37,7%** |  |
| Share of co-payment | **3,6%** |  |
| Premium of Private insurance | **4,5%** |  |
| External Resources |  | **0,1%** |
| **Total** |  | **100,0%** |

**Table S3:** Health financing sources in Tunisia 2010

Data Source: Authors ‘compilation based on Data from Ministry of Finance and MOH.

In addition, there is unbalance of financing between public health sector and private one. While the public sector deals with 80% of the population problems it receives only 20 % of the total health expenditures. On the other hand, private health facilities deal with 20% of the population and benefit of 60% of the total health expenditures.

**3. Monitoring and evaluation for UHC**

Data and data sources we used in this study case are showed in Table S4. Many institutions, in and outside MoH, are collecting a wide range of data related to Health. But, even, many Data are available and may be used to assess and evaluate progress towards UHC in Tunisia; the Health information system does not exist as an integrated tool aiming to monitor such progress since there is no defined core indicators related to UHC.

| **Indicators** | **Data Sources** | **Short description** |
| --- | --- | --- |
| Vital Data | National Institute of Statistics (NIS) | Main institution of Statistics in the country. Conduct general census, household survey and collect statistics from Ministries. |
| Routine Administrative Health Facilities Data | MoH Health Map; Annual reports of hospitals, Annual reports of Preventive Directorate of the MoH | Consistency and quality of some routine reporting systems are not usually good |
| Health Coverage indicators | Household surveys : Demographic and Health Surveys, Multiple Indicators Child Health Surveys | Conducted by NIS and UNICEF |
| MDGs indicators | NIS, INS (various publications), UNDP report (2004), World Bank (2007) | Assessment of MDGs progress from time to time using various sources |
| Health Spending and Health Expenditures | NHA, NIS, WHO | NHA 2002, 2005, NIS 2013, WHO 2010 |
| Financial Risk | NHA, NIS, MoH, authors estimation | Studied through multiple sources |
| Causes of Death in Tunisia | National Institute of Public Health | Information system launched since 1999. Statistics 2001, 2003, 2006 and 2009 |
| Human Resources of Health | “Health Map” 2011 of MOH, Medical Board Council , MoH | Information collected through related Websites |
| Tuberculosis and HIV | HIV/AIDS and Tuberculosis programs | M&E of Global Fund program |
| NCDs | Health facility information, NFHI statistics | 2011 Annual report of NFHI |
| SDHs | Household surveys : WHS, Tahina survey | WHS/Tunisia (2003), Tahina (National survey 2005) |
| **Indicators** | **Data Sources** | **Short description** |
| Vital Data | National Institute of Statistics (NIS) | Main institution of Statistics in the country. Conduct general census, household survey and collect statistics from Ministries. |
| Health Coverage indicators | Household surveys : Multiple Indicators Child Health Survey (MICS4 / 2011-2012) | Conducted by NIS and UNICEF |
| MDGs indicators | NIS, INS (various publications), UNDP report (2004), World Bank (2007) | Assessment of MDGs progress from time to time using various sources |
|  |  |  |
| Health Spending and Health Expenditures | NHA, NIS, WHO | NHA 2002, 2005, NIS 2013, WHO 2010 |
| Financial Risk | NHA, NIS, MoH, authors estimation | Studied through multiple sources |
| Causes of Death in Tunisia | National Institute of Public Health | Information system launched since 1999. Statistics 2001, 2003, 2006 and 2009 |
| Human Resources of Health | “Health Map” 2011 of MOH, Medical Board Council | Information collected through related Websites |
| Tuberculosis and HIV | HIV/AIDS and Tuberculosis programs | M&E of Global Fund program |
| NCD | Health facility information, NFHI statistics | 2011 Annual report of NFHI |
| SDH | Household surveys : WHS, Tahina survey | WHS/Tunisia (2003), Tahina (National survey 2005) |

**Table S4:** Indicators, Data and Data Sources used in the study

The Demographic Health Surveys (DHS) (the last was conducted in 2008) and the Multiple Indicator Cluster Surveys (MICS) are the principal types of surveys used as sources of information on health interventions coverage. Data coming from such national household surveys are always reliable and easy to catch. Since three decades, the country gain experience on doing health surveys and for many health indicators we could develop a trends analysis. The last health household survey (MICS) was conducted in 2011-2012 [5] and gives an interesting overview on child and maternal health state. Unfortunately, results of national household surveys give a picture of the bottom and do not allow subnational analysis.

The other main source of Health Data is the routine administrative health facilities data, which could be used to produce some indicators as service utilization or population coverage rates by human resources of health or facilities, by district and region. Estimates based on administrative data may give an annual and regular estimate which is more convenient than estimates based on surveys which are done every five years in average. Unfortunately, it’s often reproached to administrative data collected through the routine reporting systems the weaknesses of their accuracy.

The National Institute of Statistics conducted periodic household surveys to assess the Living Standards of households and provided disaggregated health expenditures data by wealth quintiles. It also records annual National Accounts statistics which could be used to produce National Health Account. But, usually there are some problems related to accuracy (over or underreporting) and discrepancies between various sources of economic information (NIS, MoH, NFHI).

It was very difficult to collect reliable Data on the users’ satisfaction or the responsiveness of the health system. Overall, the quality of services is perceived to be lower in underserved areas and in public facilities, in terms of proxy indicators and user perceptions of responsiveness.

**4. Progress towards UHC in Tunisia**

Coverage indicators

Trends of many health indicators during the last three decades, including communicable diseases occurrence, child and maternal health and institutional deliveries, show that Tunisia made a big progress in coverage indicators (Table S1). During the last 30 years, average life expectancy in Tunisia has increased. The decline of the infant mortality rate is the most important progress achieved, decreasing from 80 per thousand for the year 1980 to 16.7 per thousand currently (Figure S2).

**Figure S2:** Trends of Tunisian’s demographic indicators (1980-2012)

Data Source: Data collected from every 5 years Household surveys as Demographic and Health Surveys, Multiple Indicators Child Health Surveys

| **Indicator** | **Coverage** |
| --- | --- |
| Births delivered in a health facility | **98,5 %** |
| Births assisted by a skilled provider | **98,6 %** |
| Women receiving ANC from a skilled provider (4 ANC) | **85.1 %** |
| Married women in reproductive age using modern FP method | **50 %** |
| Family Planning Needs Unsatisfied | **7.0 %** |
| Received all basic vaccines | **78.8 %** |
| Received Measles vaccine | **94.3 %** |
| Received Hepatitis B vaccine | **95.5 %** |
| Received 3 doses of DPT vaccine | **95,5 %** |
| Received BCG vaccine | **98.2 %** |

**Table S1:** MCH Service Utilization

Data Source: Multiple Indicators Child Health Survey (2011-2012))

Table S5 shows the progress made in achieving various MDGs. Based on a linear continuation of past trends, Tunisia would seem on track to timely meeting the targets without further policy efforts. For maternal mortality, progress in achieving the target of reducing by two thirds was being insufficient and here is where the country faces one of its latest MDG challenges.

**1990**  **2000** **2015**

(or circa) (or circa) target

**MDG 4: under-five mortality rate (per 1,000 live births)**  37.3 18.4 (2007) 16.6

**MDG 5: maternal mortality rate (per 100,000 live births)** 74.8 45.6 (2008) 18.7

**MDG 7a: Access to clean water (% of population)** 75.0 97.8 (2004) 100.00

**MDG 7b: Access to basic sanitation**  59.9 (1994) 78.3 (2004) 100.00

**(% urban population)**

**Table S5:** Tunisia MDG achievement and targets for 2015

Data Source: INS (various publications), World Bank (2007), and UNDP (2004).

Non-communicable diseases

Tunisia is going to aging and the prominence of chronic conditions has increased [6, 7]. By 2020, the proportion of older persons (over 60 years) will increase to 12.5% while it was 10.1 % on 2011, and various evidence has confirmed a shift in the global burden of disease from infectious diseases and maternal and child health conditions to non-communicable and chronic diseases (NCDs) [8]. The top three causes of mortality are ischemic heart disease, cancers and respiratory tract diseases (Table S6). New demands will be made and need to be address (Figure S1) but, while the health system tries to secure availability of medicines in order to treat the most frequent non-communicable disease, as Diabetes or Hypertension, there is a greater likelihood that shortages would be experienced in all of health facilities [9].

**Figure S1:** Trends of Chronic Diseases

Data Source: Statistics of National Health Insurance Fund (NHIF/CNAM); 2007-2011

| **Causes of Death** | **2001** | **2003** | **2006** | **2009** |
| --- | --- | --- | --- | --- |
|  | % | % | % | % |
| **Circulatory System Diseases** | 28.2 | 28.2 | 28.9 | **28.9** |
| **Cancers** | 15.4 | 15.1 | 16.5 | **16.1** |
| **Respiratory System Diseases** | 9.1 | 11.4 | 9.5 | **11.2** |
| **Metabolic and Endocrin Diseases** | 8.6 | 9.7 | 9.9 | **10.2** |
| **Prenatal Period Pathology** | 9.4 | 8.0 | 6.8 | **6.6** |
| **Trauma and Empoisonning** | 3.4 | 3.1 | 3.1 | **3.6** |
| **Communicable Diseases** | 3.5 | 2.9 | 2.8 | **3.0** |
| **Violent Deaths** | 5.1 | 3.8 | 3.8 | **2.9** |

**Table S6**: Most frequent causes of Death in Tunisia

Data Source: NIS

Considering risk factors of non-communicable diseases, tobacco consumption is a major risk factor for adults aged 25-59 years, especially men, but also increasingly for young women [7]. Both men and women exhibit high prevalence of obesity, high cholesterol, and high blood pressure—a group of risk factors with a common pathway to premature cardiovascular disease and mortality. Given the link between obesity and diabetes, hypertension, heart disease, stroke, and many other chronic diseases, this has serious consequences on future health spending.

Financial risk protection

Since 1960, Tunisia had established a social health protection strategy with a two-tiered system of health insurance and subsidized or free care. Based on the principles of assistance and insurance, it’s funded through employee contributions and government-subsidized coverage for those who are poor and pro-poor population.

For poor and pro-poor population, there are two public medical aid schemes to access to health care in governmental facilities: the free healthcare scheme, which covers households being defined according to local poverty line and regional quotas, and the reduced-fee plan, which covers whose individual monthly income falls below the minimum wage rate. Poor and pro-poor households covered by these schemes (free care and reduced-tariffs) represent about 24% of the whole population. The poorest are exempt from all fees, while others who are eligible for a reduced-tariffs scale.

Currently, the NFHI is the main health insurance mechanism in Tunisia. It has been launched in 2006 under a reform that has merged several insurance schemes previously covering certain professional groups. It is a mandated social insurance scheme for workers in the public and formal private sectors and their dependents, financed by employee and employer contributions and covering 68 % of the population. Following the 2006 reform, coverage was extended to include inpatient and outpatient services provided by the private sector, although reimbursement mechanisms limit coverage for a predetermined list of chronic illnesses and surgical interventions and remain subject to an annual expenditure capita per household.

Although this steady progress of financial risks protection, gaps remain. Despite the existence of these both insurance schemes, insurance coverage in Tunisia remains incomplete while 8 to 10% (near one million people) are not covered. In addition, there is consensus that the public medical aid schemes for poor and pro-poor population are inefficient, and puts a big strain on public hospitals’ budget. The Tunisian population has also a higher average household spending on healthcare; out of pocket expenditure is as high as 45.8 % of total health expenditure in 2010 data (Table S3). Tunisian citizens in the low income bracket are not, therefore, guaranteed access to health care they need.

Thus, financing gaps are identified as a remaining barrier to reaching universal coverage in the country. Figure S3 displays the magnitude of the exposition to catastrophic health expenditures and the share of these catastrophic expenditures by country region and quintile. The Great Tunis, Northeast, and Centre east were similar in this respect (around 4%). The Western regions (north, center and south) showed a much higher proportion of catastrophic spending for the three lowest wealth quintiles. In all regions the same trend of increasing catastrophic expenditures with lowest socio-economic position was observed. The proportion of households exposed to CHE is concentrated amongst the poorest quintile of households. Abou Zaineh, Arfa C. and al [10] shows that 0.32 % of population full in poverty due to CHE using the national extreme poverty line (0.86 US$ per day) while it rises to 0.66 % when we consider the World Bank extreme poverty line (1.08 US$ per day).

**Figure S3:** Catastrophic health expenditure (40% of total household non-food expenditures) by country socioeconomic regions with reference to quintile.

Data Source: 2010 National Survey of budget, consumption and living standards

Equity issues

Despite the majors improvements achieved by the Health system during the past 50 years, many evidences point out the persisting gaps between the coastal areas and those of western part of Tunisia, which reflects social inequalities and health inequities. According to the MoH Health Map 2011, 95% of people have acceptable geographic access to primary care facilities (less than 5 km distance), but at the coastal areas, the institutional health facilities are more furnished than those of western part of the country where the number of specialists and doctors, the quality of equipment and the coverage of services are all much lower (Figures S4, S5). In addition, the infant mortality rate is two times higher in rural area (25 %0) than in urban area (12 %0) as well as the under 5 mortality rate which is too higher in rural area (26 %0 vs 15 %0).

**Figure S4:** Access to General Hospital by governorates

Data Source: MOH, Health Map 2011. http://www.santetunisie.rns.tn/msp/msp.html


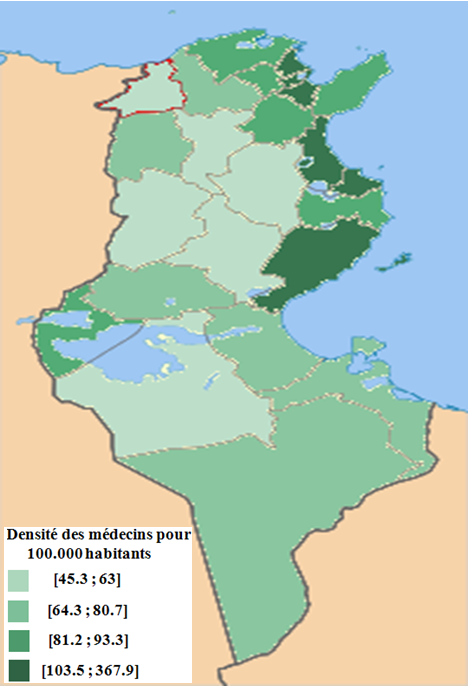


**Figure S5:** Density of Physicians by governorate

Data Source: Data Sources of Figure S5: MOH, Health Map 2011. http://www.santetunisie.rns.tn/msp/msp.html

It is well admitted that remaining inequities based on income and degrees of urbanization in Tunisia have contributed to recent revolution. The urbanization rate is highest in the Tunis district (92.1%) and in the eastern central region (72.6%), which are the most economically and socially developed areas. Only the western central and north-western regions remain predominantly rural (with urbanization rates of 33% and 37.8%, respectively) and under-developed areas.

The Tunisian government has acknowledged that progress at the national level is not always matched at the sub-regional level where disparities do exist. While some regions in the country have experienced remarkable strides in development, others are still lagging behind according to the UNDP report available (MDGs, UNDP, 2004).

Comparative assessment

Unlike many of other North African countries, Tunisia has no malaria and low rates of HIV/AIDS. It has a tuberculosis rate that is one-quarter that of Morocco and one of the lower maternal mortality rate in the North African countries [11,12]. The life expectancy is around 75 years, the higher one of the region. Nearly 90% of Tunisia’s citizens have access to health insurance that provides a relatively good level of basic services—a higher coverage rate than in Algeria and Morocco. In addition Tunisia’s health system had built a robust infrastructure for primary healthcare and a strong medical education system. Manpower indicators are better than Morocco and Hospital bed ratio is better than both countries.

**5. Conclusions and recommendations**

Tunisia made a significant progress to achieve UHC. However, the Health system faces currently some obstacles: The remaining gap of access to health services between poor populations and those with better living conditions; the emergence of chronic and non-communicable diseases which require lot resources to make available needed treatments; the unbalanced development of the health system with a growing private sector contrasting with an institutional health sector less efficient; and the remaining high level of Out-of-pocket expenses.

To extend universal health coverage to those who live in rural and underdeveloped areas and less covered is the big challenge for the future in Tunisia considering the current access gaps and the growing demands due to the increased burden of non-communicable diseases which need to secure greater availability of medicines and drugs. The increased prevalence of obesity, diabetes, hypertension, heart disease and stroke recorded during the last two decades has serious consequences on future health spending and financial risk protection, mainly for poor and pro-poor population.

Regarding financial risk protection and health insurance, 8 to 10 % of the population is not covered against the health risks. It mainly concerns those who are unemployed, seasonal and occasional workers. All of them have to pay, by themselves or should be helped by their family members, any medical fees they need. For such vulnerable persons, the enjoyment of the highest attainable standard of health, one of the fundamental rights of every human, is not achievable.

The high households’ contribution, about 45 % of the total health expenditures, contrasts with the low contribution of the NIHF and the public medical aid schemes supported by the government, which means health iniquity and social injustice. The rising of out of pocket payment in Tunisia is the result of the rapid growing of the private sector, the failure of the 2006 health insurance reform and the low total government spending on health. According to Abuja Declaration, public funding for healthcare shall be 15% of the government budgets. Its share in Tunisia remains far from that target since it was declined from 9 to 6 % over the past decade.

Given the high level of the household catastrophic expenditure, particularly among the poorest quintile of population, there is an urgent need to revisit the current health financing strategy which places the burden of payment on households. Instead, the government should identify ways of financing health care that rely less on individual payments at point of use, and allow for a greater degree of risk sharing and other forms of risk protection, particularly for the poorest that are often covered by medical assistance scheme. It’s seems suitable expanding the existing national health insurance scheme, to include more groups of people and benefits, and targeted subsidies or payments to reach the poor, by for example merging the medical assistance schemes into NFHI.

In Tunisia, it’s well admitted that the achievement of the universal health coverage for the whole population is being constrained mainly by gaps in financing. Tunisian health financing is a mixed and fragmented system due to the existing multiple mechanisms of financing and coverage. The system lacks clear and consistent funding strategy, weak solidarity, and high cost of the overall management of the health system. Consequently, there’s a need to identify and discuss priority challenges and approaches to development and implementation of health financing policies oriented to universal coverage.

The other challenge of implementing universal coverage in Tunisia is related to the balance between public and private sector. It depends on how the public-private mix of healthcare delivery would be. Under the scenario where the private sector becomes the dominant provider of care, the health expenditures should grow. If managed properly, we would have a mixed scheme with a public system efficient enough to deliver a basic package and a private system that would have the quality to provide some extra care for those who could pay a bit more. This scenario will be one of the most challenging to enact within the given timeline, given high level of out-of-pocket health payments.

The current UHC assessment showed that some gaps in implementation of UHC in Tunisia remain. The current transitional context of Tunisia, after revolution, seems to be like good politically to push enough UHC to commit decision-makers and gain public support. Also, civil society organizations (CSOs) may be involved in progressive realization of UHC. For example, in determining service packages and methods of provision of health care for the future, which need, through a social dialogue, to introduce news methods and mechanisms to reinforce the potential role of CSOs in tracking progress towards UHC.

Tunisia needs to develop an appropriate monitoring and evaluation system to follow UHC progress in order to collect Data in many areas for which there are no good indicators as NCD treatment coverage, quality of health care and private sector use. To monitor interventions coverage, the household surveys are considered, right now, as the most indicated tools. However, they should make possible subnational analysis which is very important as the extent to which UHC can be achieved depends on how well these subnational differences can be addressed as health inequity remains an important issue between governorates.

For chronic conditions, even some recent surveys on prevalence and risk factors of NCDs were conducted and give interesting results; we need to conduct new household surveys as monitoring system and surveillance of effective coverage of NCDs treatment. It’s also needed to help and encourage the implementation of the hospital information system. Such tool could be useful and helpful to produce some indicators related to injuries, cancer and others NCDs.

The future UHC M&E system in Tunisia should be an integrated tool based on periodic household surveys focusing, at least, on interventions coverage and effective coverage of NCDs treatment, Data related to injuries and others chronic conditions and geographical distribution of human resources. Regarding financial risk protection monitoring, the incidence of impoverishment due to out-of-pocket payments are not commonly used and not identified as a big concern of decision-makers. Since such data are available, we need to develop skills to use and interpret health financing data to build capacity to monitor such UHC issue.

| **Box S1: Challenges and Recommendations**   \| **Challenges** \| **Recommendations** \| \| --- \| --- \| \| 1. Gap of access to health services for poor populations and remote rural areas \| More HRH and equipment for remote and under covered areas \| \| 2. Lack of resources to make available needed treatments of NCDs in PH facilities (PHF) \| Rise Budget of NCDs Treatment for PHF and improve drug management through PHF \| \| 3. Increased prevalence of obesity, diabetes, hypertension, heart disease and stroke \| Implement efficient strategy to track NCDs risk factors \| \| 4. Lack of quality of care among PHF \| Rise the % of Government Budget for Healthcare to 15% and improve training for  Health staff \| \| 5. Rapid growing of private sector without good regulation \| Encourage a public-private mix of healthcare to deliver both the same basic package of services with the same quality and cost \| \| 6. High level of Out-of-pocket expenses \| Identify ways of financing health care that rely less on individual payments at point of use, allow greater degree of risk sharing and create other forms of risk protection \| \| 7. About 10 % of the population is not covered against the health risks \| Extend existing national health insurance scheme to include not covered people \| \| 8. The health financing system lacks clear and consistent funding strategy, weak solidarity, and high cost of the overall management of the health system \| Need to identify and discuss priority challenges and approaches to development and implementation of health financing policies oriented to universal coverage \| \| 9. Lack of Data in many areas for which there are no good indicators as NCDs treatment coverage, quality of health care and private sector use. \| Conduct new type of household surveys as monitoring system and surveillance to measure such issues \| \| 10. Lack of Data for other chronic conditions and Injuries \| Encourage the implementation of the hospital information system tool \| \| 11. The Health information system does not exist as an integrated tool aiming to monitor UHC progress and there is no defined core indicators related to UHC \| The future UHC M&E system in Tunisia should be an integrated tool based on periodic household surveys focusing, at least, on interventions coverage and effective coverage of NCDs treatment, Data related to injuries and others chronic conditions and geographical distribution of human resources \| \|  \|  \| |
| --- | --- | --- | --- | --- | --- | --- | --- | --- | --- | --- | --- | --- | --- | --- | --- | --- | --- | --- | --- | --- | --- | --- | --- | --- | --- | --- |

**References**

1. Editorial. (2012) Universal health coverage: the third global health transition? Available: www.thelancet.com Vol 380
2. Hermassi H. (2013) Tunisian Revolution and Regional Imbalance. Journal of Management and Business Studies 2(2): 80-84. Available: http://garj.org/garjmbs/index.htm
3. National Institute of statistics. Available: http://www.ins.nat.tn/indexen.php
4. Health Map 2011. Available: http://www.santetunisie.rns.tn/msp/msp.html
5. Anonymes. NIS/UNICEF. (2013) Multiple Indicator Cluster Surveys (MICS) 2011-2012. Available: http://www.ins.nat.tn/
6. Ben Romdhane H. (2006) Transition Epidemiological and Health Impact North Africa.
7. Ben Romdhane H and Grenier FR. (2009) Social determinants of health in Tunisia: the case-analysis of Ariana. International Journal for Equity in Health.
8. Ghanem H. (2011) The need for capacity building to prevent chronic diseases in North Africa and the Middle East; Eastern Mediterranean health Journal 17(7): 630-2
9. Ben Salem K et al. (2000) Les médicaments en première ligne: disponibilité des médicaments et prescription à Monastir (Tunisie). Revue de santé de la méditerranée orientale, 6 : 2-3
10. Abu-Zaineh M, Chokri A, Ventelou B, Ben Romdhane H and Moatti JP. (2013) Fairness in healthcare finance and delivery: what about Tunisia? Health Policy and Planning. Advance Access.
11. Chaoui F, Legros M, Achour N, Benbrahim NF, Grangaud JP. (2012) Les pays du Maghreb, des États en transition sanitaire. ; Les systèmes de santé en Algérie, Maroc et Tunisie : Défis nationaux et enjeux partagés. Les Notes IPEMED. Available: http://www.ipemed.coop/fr/publications
12. Anonymes. (2013) Fairness and accountability: Engaging in health systems in the Middle East and North Africa. Available: www.worldbank.org/mena/health-strategy.
